# Implications of uremic cardiomyopathy for the practicing clinician: an educational review

**DOI:** 10.1007/s10741-023-10318-1

**Published:** 2023-05-12

**Authors:** Hiroaki Hiraiwa, Daisuke Kasugai, Takahiro Okumura, Toyoaki Murohara

**Affiliations:** 1 Department of Cardiology, Nagoya University Graduate School of Medicine, 65 Tsurumai-cho, Showa-ku, Nagoya, 466-8550 Japan; 2 Department of Emergency and Critical Care Medicine, Nagoya University Graduate School of Medicine, 65 Tsurumai-cho, Showa-ku, Nagoya, 466-8550, Japan

**Keywords:** Heart failure, Kidney disease, Uremic cardiomyopathy, Left ventricular hypertrophy, Diastolic dysfunction

## Abstract

Studies over recent years have redeveloped our understanding of uremic cardiomyopathy, defined as left ventricular hypertrophy, congestive heart failure, and associated cardiac hypertrophy plus other abnormalities that result from chronic kidney disease and are often the cause of death in affected patients. Definitions of uremic cardiomyopathy have conflicted and overlapped over the decades, complicating the body of published evidence, and making comparison difficult. New and continuing research into potential risk factors, including uremic toxins, anemia, hypervolemia, oxidative stress, inflammation, and insulin resistance, indicates the increasing interest in illuminating the pathways that lead to UC and thereby identifying potential targets for intervention. Indeed, our developing understanding of the mechanisms of UC has opened new frontiers in research, promising novel approaches to diagnosis, prognosis, treatment, and management. This educational review highlights advances in the field of uremic cardiomyopathy and how they may become applicable in practice by clinicians. Pathways to optimal treatment with current modalities (with hemodialysis and angiotensin-converting enzyme inhibitors) will be described, along with proposed steps to be taken in research to allow evidence-based integration of developing investigational therapies.

## Introduction

Cardiovascular disease is the leading cause of death in patients with chronic kidney disease (CKD), ahead of even end-stage renal disease (ESRD) [1]. While the contribution of coronary artery disease to this burden of mortality is well established [2], research beginning in the 1970s and 1980s identified left ventricular hypertrophy (LVH) and congestive heart failure (CHF) as more significant causes of death in this patient group [3]. These conditions, along with associated cardiac hypertrophy and abnormalities resulting from CKD such as left ventricular dilatation and systolic and diastolic dysfunction, are often described under the term uremic cardiomyopathy (UC), although definitions have conflicted and overlapped over the decades, complicating the body of published evidence and making comparison difficult.

These conflicting definitions have made it challenging to precisely define the size of this patient population worldwide, but reports from the USA estimate that 2 million people in that country alone will require dialysis for ESRD by 2030 [4] and that LVH is found in some 70% of patients with ESRD [5, 6].

Among those patients with ESRD, cardiovascular disease is the cause of approximately half of deaths [6], and an analysis of over 1 million patients revealed an independent graded association between reduced glomerular filtration rate (GFR) and risk of cardiovascular events and death [7]. However, evidence indicates that this level of cardiovascular death is largely secondary to LVH and CHF rather than to atherosclerosis [4, 5]. The primary manifestation and hallmark of UC is LVH [8], often accompanied by increased ventricular thickness, arterial stiffening, coronary atherosclerosis, and coronary artery calcification. Clinical symptoms, while similar to those resulting from critical coronary artery disease, arise in patients with UC because of the reduced coronary reserve that results from adaptive (and eventually maladaptive) LVH [9].

Established risk factors associated with the development of UC include hypertension, hyperlipidemia, and diabetes, which are also well-known risk factors for atherosclerotic disease [10]. UC also appears to be associated with certain other and related risk factors for cardiovascular disease (e.g., increased age, obesity/sedentary lifestyle, tobacco use) that suggest a future global increase in UC incidence, and likely in CKD incidence overall, will accompany the aging of populations and Westernization of lifestyles.

However, other more specific potential risk factors for UC have been identified, including uremic toxins, anemia, hypervolemia, oxidative stress, inflammation, insulin resistance, and CKD-mineral and bone disorder (MBD) [9]. Continuing research in these areas indicates the increasing interest in illuminating the pathways that lead to UC and thereby identifying potential targets for intervention. Indeed, our developing understanding of the mechanisms of UC has opened new frontiers in research, promising novel approaches to diagnosis, prognosis, treatment, and management.

**Fig. 1:**
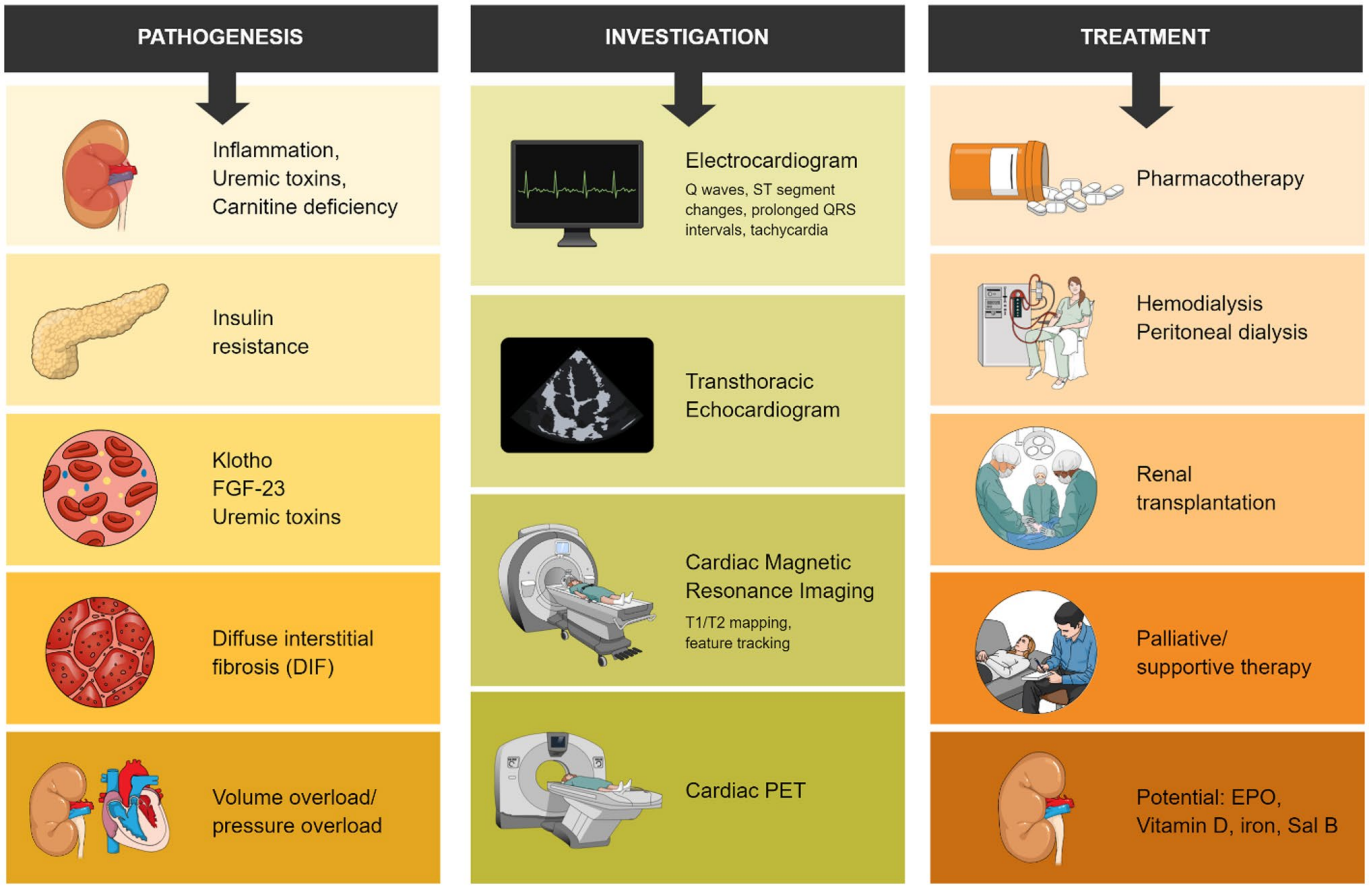
Current understanding of uremic cardiomyopathy

The topics covered in this review are aimed at providing an overall understanding of UC (Fig. 1), particularly advances in the field of UC that are applicable in practice for clinicians. Pathways to optimal treatment with current modalities (including hemodialysis and angiotensin-converting enzyme (ACE) inhibitors) will also be described, alongside the integration of developing investigational therapies.

## Current knowledge

### Clinical background

UC was first described by Bailey et al. in 1967 [11]. From that time on, it was described as characterized by LVH, diffuse interstitial fibrosis (DIF), focal scarring, and systolic and diastolic dysfunction. In the intervening decades, UC was considered a severe cardiomyopathy associated with ESRD and characterized by severe functional abnormalities that could be reversed by renal transplantation. This theoretical framework, however, has been increasingly replaced by an understanding of UC as a clinical phenotype of cardiac disease that accompanies CKD, with a multitude of contributing factors and best characterized as diastolic dysfunction seen in conjunction with LVH and fibrosis [12].

### Pathogenesis

The definition of a typical time duration for the development of UC after the onset of CKD is difficult because of the lack of a firm definition or threshold for UC. However, the disease process itself becomes evident in the early stages of CKD, at relatively preserved estimated glomerular filtration rates [13].

As the field progresses, researchers should take care to identify any potential differential or atypical patient subsets in whom UC may develop differently. In children and adolescents with CKD, for example, there is typically no clinical background of pre-existing cardiac disease, although the risk of cardiovascular death is similar to that in adults with CKD [14]. The implication is that the high cardiac death rate is not fully explained by traditional cardiac risk factors, with much of the increase attributable to uremia-related risk factors. This assumption is given credence by the lower mortality observed in young patients after kidney transplant versus with those receiving continued dialysis [14]. Regardless, the development of CVD in children with CKD is clearly influenced by multiple factors, meaning that prevention efforts must focus on the identification of modifiable risk factors [15]. The picture is further complicated by the prominence of congenital (non-modifiable) anomalies of the kidney and urinary tract, which are recognized as the leading cause of ESRD in children and present a different pathophysiology from adult-onset ESRD [16]. To date in this subset of patients with CKD, there has been no high-quality evaluation of the associations between childhood CVD and later cardiac mortality, and interventional studies controlling modifiable CV risk have not been reported; no standard guidelines for screening or treatment in this population are yet available. Clearly, CVD in the setting of children with CKD remains poorly understood, with notable gaps in the research remaining to be filled.

Regardless of patient type or stage of development, the pathogenesis of UC is clearly multifactorial, involving multiple pathways including circulating uremic toxins, mineral metabolism, insulin resistance, and eventually hemodynamic overload. These pathways contribute to the increased left ventricular (LV) mass common in CKD and attributable to both myocyte hypertrophy and an expansion of the interstitial space caused by DIF [12].

A deficiency in carnitine, an important factor in the oxidation of fatty acids, is postulated to play a role in insulin resistance and the development of UC, on the basis of observations in patients undergoing dialysis [17]. Insulin resistance is an independent risk factor for CVD in patients with CKD, believed to be related to interruption of the intracellular insulin pathway that occurs as a result of increased angiotensin II, inflammation, metabolic acidosis, and uremic toxins [18].

The process of cardiac metabolic remodeling in patients with CKD is characterized by various forms of cardiac metabolic maladaptation including altered mitochondrial function, myocardial substrate utilization, metabolic transporter function and expression, and impaired insulin response and phosphoinositide-3 kinase-AKT signaling, leading to impaired cardiac function [19]. These maladaptations are mediated via the interplay between contributions of volume overload and pressure overload: LV pressure overload mediates hypertrophy by increasing LV wall thickness with minimal change in chamber size, whereas LV volume overload results in increased chamber size but normal LV wall thickness [20].

Uremic toxins, by definition uremic retention solutes that impede normal organ function, represent another pathway leading to UC and include P-cresyl sulfate (pCS), β2-microglobulin, indoxyl sulfate, and homocysteine [21]. Many of these retained compounds are not effectively removed or eliminated by current methods of dialysis.

Additionally, certain monocyte/macrophage subsets have been shown to be positively or negatively associated with cardiovascular calcification, an important predictor of CVD, in patients with CKD [22]. The influence of uremic toxins (as well as the treatment a patient may be undergoing for CKD) may affect the phenotype and function of these monocyte/macrophage subsets, resulting in osteogenic transformation and mineralization of the vascular cells.

One of the most promising areas of understanding UC is the recent progress in understanding the role of low serum levels of Klotho and elevated serum levels of FGF-23, both regulators of phosphate metabolism, in the development of LVH [23]. In patients with CKD, deficiencies in renal excretory capacity typically lead to massive increases in FGF-23 to compensate and maintain phosphate levels. These levels have been identified as being associated with CVD and mortality in a dose-dependent manner [24]. Klotho, a single-pass transmembrane protein expressed in the kidney, is a co-receptor for FGF-23 that mediates the effects of FGF-23 in phosphate regulation; some factors that are elevated in kidney dysfunction (angiotensin II and inflammatory cytokines) downregulate Klotho expression in the kidney. There remains a lack of consensus over whether Klotho and FGF-23 act on an axis or whether Klotho is protective independently of FGF-23 levels [25], but this area of research represents a potential therapeutic target for the treatment of UC, as described below.

Importantly, none of the established treatments for CKD directly targets the metabolism of the uremic heart, implying a potential area for the development of new treatments.

## Screening and diagnosis

Because of the heterogeneous nature and involvement of multiple systems in UC, current clinical methods for its diagnosis involve multiple modalities.

Electrocardiogram (ECG) is the most widely available, least invasive, and least expensive method for assessing LV dysfunction and identifying volume and pressure abnormalities in patients with CKD. ECG changes that are characteristic or indicative of UC include the presence of Q waves, ST segment changes, prolonged QRS intervals, and tachycardia [26]. Associations between QT interval, spatial QRS-T angle, signal-averaged ECG, heart rate variability, and T-wave alternans and mortality have also been reported in patients undergoing dialysis [27], although the causal direction of these associations remains unclear. This modality is also of value in differentiating UC from other forms of cardiomyopathy.

As summarized in Table 1 [33], cardiac imaging in patients with CKD can involve multiple modalities, including echocardiography, cardiac magnetic resonance imaging (MRI), and cardiac positron emission tomography (PET).

**Table 1:** Summary of current cardiac imaging modalities used in UC

Modality	Use in UC	Characteristic findings in UC	Advantages and limitations	References
Echocardiography	Evaluation of function and structure of myocardium; evaluation of cardiac valves	Volume and pressure abnormalities; increased LV mass index; presence and severity of LVH	Cost-effective; noninvasive; portable; can accurately assess for hypertrophy of myocardium Operator dependent; prone to inaccuracy due to measurements being derived; no information on epicardial artery or microvascular disease; no information on interstitial tissue	Arrigo et al. [28] Foley et al. [29]
Cardiac MRI	Evaluation of myocardial structure and function, cardiac valves, myocardial interstitium, and coronary artery flow	Myocardial fibrosis, myocardial edema	More accurate and reproducible than echocardiography; higher imaging quality compared with echocardiography; gold standard of cardiac imaging More expensive; less accessible; use of gadolinium-based contrast associated with nephrogenic systemic fibrosis	Arcari et al. [30] McIntyre et al. [31]
PET	Evaluation of myocardial perfusion, micro- and macro-vasculature, and left ventricular function	Ischemia, infarction, inflammation	Reliable assessment of ischemia; allows for evaluation of microvasculature Variability of radiotracer uptake contributes to variations in results; little research into distribution of radiotracer uptake in kidney failure	Lau et al. [32]

Adapted from Kott et al. [33]. CC-BY-NC.

*MRI* magnetic resonance imaging, *PET* positron emission tomography, *UC* uremic cardiomyopathy, *LV* left ventricular, *LVH* left ventricular hypertrophy

Transthoracic echocardiography (TTE) is a readily available, inexpensive, and noninvasive modality offering detailed observation of cardiac structures and potential abnormalities, particularly LVH. TTE is a well-established method to assess LV mass, commonly used to provide prognostic information or as an endpoint in studies of CVD risk. However, these methods involve indirect calculation of LV mass rather than direct observation of the actual measurements, and a large meta-analysis showed no clear association between changes in LV mass and CV mortality in patients with CKD [34], limiting the utility of such measures as a surrogate endpoint and highlighting the need for further imaging for assessment.

Cardiac computed tomography (CT) scanning can be used in UC, for example, to define areas of myocardial fibrosis [35]. This is of particular interest because the extent of fibrosis has been shown to be a strong predictor of CV death [31].

Cardiac MRI (cMRI) can be used to provide information on multiple cardiac structural and functional parameters including coronary arterial flow, perfusion, myocardial scarring, and interstitial fibrosis [36]. cMRI is of particular use in the diagnosis of UC because of its reproducibility and significantly greater quantitative information allowing for smaller sample sizes versus TTE. As a result, cMRI has become the standard for cardiac imaging where available and practical.

Additionally, there has been some research into the use of PET in the setting of CKD to evaluate myocardial perfusion and identify infarcted areas, as well as to assess coronary flow reserve [37] and areas of viability and inflammation [32].

Each of these imaging methods has some limitations in diagnosing or predicting UC, particularly in differentiating UC from other cardiomyopathies. However, these methods continue to evolve and become more practical and specific.

### New and potential methods for the diagnosis of UC

Continuing advances in imaging have led to promising techniques for improved diagnosis of UC. For example, the fibrotic process can now be observed on cardiac MRI by T1 mapping, a technique that quantifies the relaxation time of protons on inversion recovery prepared images (longitudinal relaxation (T1) times) by using analytical expression of image-based signal intensities [5]. Fibrotic areas show greater accumulation of gadolinium, appearing as an area of high-intensity signal with a shorter T1 time compared with adjacent normal tissue. A comparison of myocardial T1 and T2 times before and after kidney transplantation suggests that T1 times are indeed a stable measure of DIF [38].

**Fig. 2:**
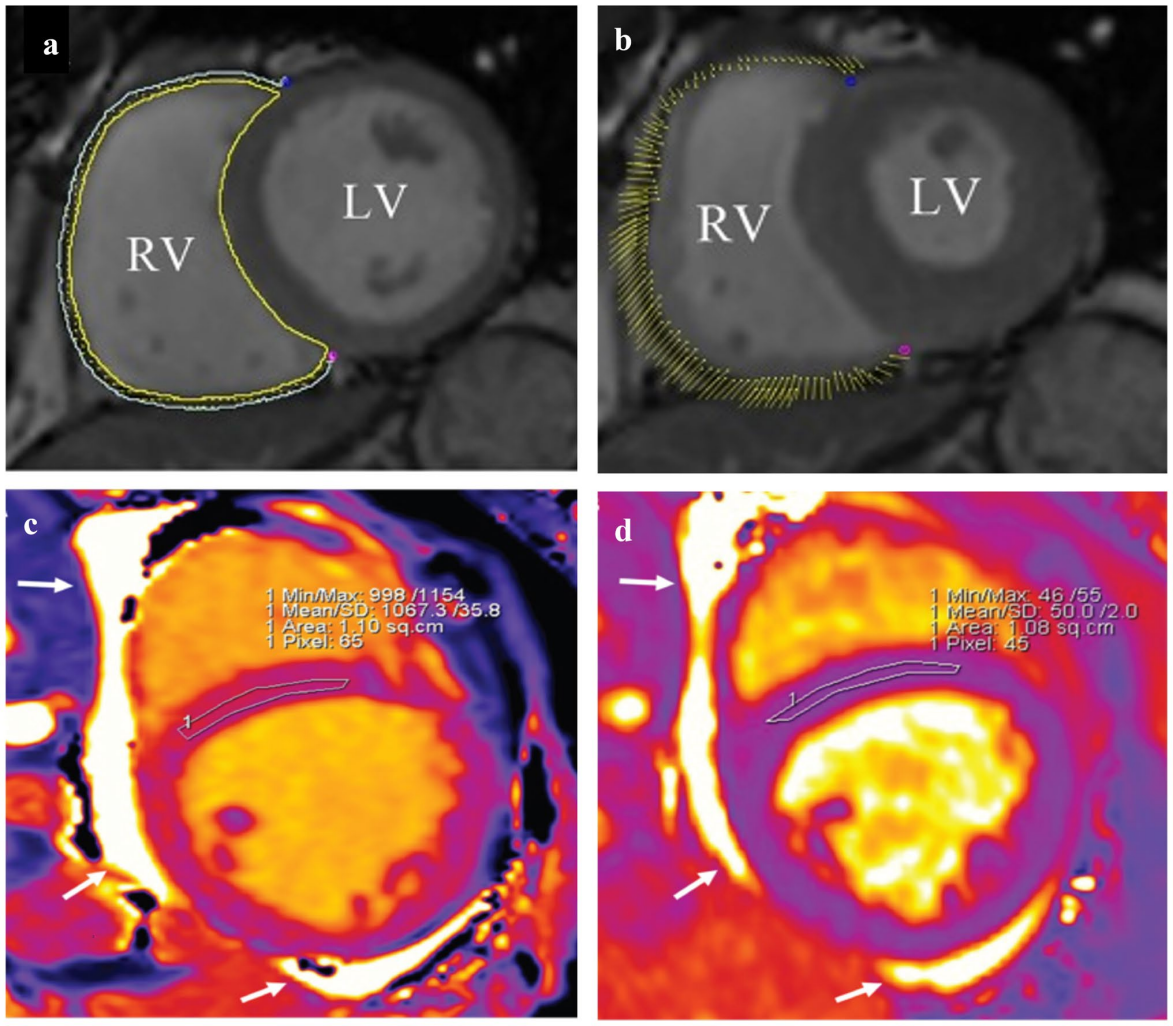
**a**, **b** Cardiac MRI images in a patient with normal heart function. This image also shows feature tracking using cmr42 (Circle Cardiovascular Imaging Inc., Calgary, Canada) in short-axis cine images at the end-diastole (**a**) and end-systole (**b**). The yellow and cyan curves delineate the endocardial and epicardial contours, respectively. The yellow dots represent the right ventricle myocardial voxel points, and the short yellow line on the images shows the tracking of the ventricle myocardial voxel points. Adapted from Hu et al. [56]. CC-BY 4.0. **c**, **d** Cardiac MRI images in a patient with stage 4 chronic kidney disease, short-axis, mid-slice, region of interest conservatively drawn in the mid-septum. Native T1 is increased at 1067 ms (**c**). The T2 value is also slightly increased at 50 ms (**d**). The left ventricle showed eccentric hypertrophy and dilation; no late gadolinium enhancement was detected. The examination was performed with a 1.5-T scanner (Siemens Aera); in-center cut-offs for normality are 995 ms (native T1) and 49 ms (T2). White arrows indicate pericardial effusion. Adapted from Arcari et al. [30]. CC-BY-NC 4.0.

These cardiac MRI T1 and T2 (transverse relaxation time) mapping techniques can be used to characterize myocardial involvement in CKD, with higher scores related to worsening clinical status and myocardial damage [39] (Fig. 2). Importantly, some native T1 and T2 mapping techniques may be performed without the need for gadolinium, the repeated use of which may represent a patient safety issue. These findings obtained via techniques could therefore potentially be used as surrogate endpoints to gauge the efficacy of interventions.

In other developments in cardiac MRI, the feature-tracking analysis technique involves a post-processing algorithm that uses a block-matching approach to assess the movement of specific anatomical features and identify longitudinal strain throughout the course of the diastole and systole [40]. A newer imaging technique analogous to cMRI feature tracking is speckle-tracking echocardiography (STE), which tracks the movement of specific echoes within the myocardium during systole to identify deformations and abnormal systolic myocardial LV function in patients with CKD and normal LV ejection fraction (LVEF) [41] and to evaluate LV global longitudinal strain.

New and more useful quantification measures are being developed along with the abovementioned novel imaging techniques. For example, global longitudinal strain (measured via conventional 2-dimensional echocardiography) has been reported to be a more sensitive predictor of myocardial dysfunction in ESRD compared with LVEF [42].

One obvious step to further advance screening and diagnosis procedures in the clinic would be to identify and quantify risk factors for UC and to apply that information to develop a predictive scoring system. This will be challenging because, compared with other cardiomyopathies, there are probably few findings that are specific to UC. To date, unfortunately, not enough is known about the use of existing or emerging diagnostic biomarkers or other means of detecting asymptomatic patients who may be at high risk for UC. The establishment of specific imaging methods should also be a priority. Echocardiography and MRI are the most promising noninvasive modalities.

In addition to developing the means to diagnose UC, in general practice, it will be important to detect ischemic changes early, based on their association with CKD, using ECG, echocardiography, chest X-ray, or coronary artery CT. This means that cardiac function should be screened and followed up from the early stages of CKD. In age-related decline in cardiac function, diastolic dysfunction is generally accepted to appear prior to systolic dysfunction. Echocardiography is the preferred modality for this identification, but as mentioned above, ischemic changes may also be detected by ECG. For patients who are already showing typical symptoms of cardiomyopathy, it may be too late to implement interventions that could change the clinical course.

Significant clinical experience has shown that UC is closely associated with CKD and that cardiac function declines over time in patients with CKD. The most common cause of this decline is ischemic heart disease. Therefore, if coronary artery stenosis is observed, revascularization procedures such as percutaneous coronary intervention (PCI) or coronary artery bypass grafting (CABG) may be performed, with resulting improvement in cardiac function. However, myocardial injury in UC is likely to extend beyond myocardial ischemia to endothelial damage in the microvasculature and cellular-level damage, as well as to the development of myocardial fibrosis. There is sufficient overlap between ischemic cardiomyopathy and UC to make it clinically difficult to distinguish between these two conditions, although it is important to treat UC when it cannot be explained as ischemic cardiomyopathy. To date, however, there is no specific treatment for UC, and the utility of current treatments may be insufficient at this point. Clarification in this area will be an important future issue for both diagnosis and treatment.

In terms of advances that can easily and immediately be adapted to the clinic, it is important to note that in no area related to UC does there already exist a sufficient evidence base to adopt new procedures based on current knowledge. For this reason, filling the knowledge gaps will be of critical importance.

## Disease management

This section will discuss the application of new findings in the therapeutic management of UC, the monitoring of disease progression, and the overall efficacy of available treatment. Current available care options, the limitations thereof, and the potential of new therapeutics are presented in Table 2.

**Table 2:** Summary of strengths and limitations of current and potential treatment modalities

Modality	Use	Advantages	Limitations
Current pharmacotherapy: ACE inhibitors ARBs Angiotensin receptor/neprilysin inhibitors SGLT2 inhibitors Beta blockers Mineralocorticoid receptor antagonists	As dictated by heart failure treatment guidelines in the use of medications	As a treatment for heart failure, evidence for cardioprotective effects and improved prognosis is well established	Treatment not specific to UC Some drugs are difficult to use in cases of CKD and reduced renal function. However, some drugs have been shown to be renoprotective
Hemodialysis	Used for cases of acute and chronic renal failure	The usefulness of the short-term therapeutic effect is well established	No evidence of improved cardiac prognosis (prognosis of UC)
Peritoneal dialysis	Used for cases of acute and chronic renal failure	Less stressful on the body compared with standard hemodialysis	No evidence of improved cardiac prognosis (prognosis of UC)
Renal transplantation	Alternative treatment for noncompensated chronic renal failure	Widely utilized in clinical practice and established as a treatment method	Evidence of improved cardiac prognosis does not appear to be established (especially for UC)
Apheresis	Used for cases of acute and chronic renal failure	Usefulness for a short-term therapeutic effect	No evidence of improved cardiac prognosis (prognosis of UC)

*ACE* angiotensin-converting enzyme, *ARB* angiotensin receptor blocker, *SGLT2* sodium-glucose co-transporter-2, *UC* uremic cardiomyopathy, *CKD* chronic kidney disease

Current options for pharmacotherapy for patients with UC include many therapies used for heart failure in general. In particular, these include ACE inhibitors and angiotensin receptor blockers, which have been shown to reduce the progression of renal and vascular damage in patients with CKD [43]; carvedilol, which improves LV end-diastolic pressure, LV end-systolic volume, and LVF and appears to reduce symptoms and hospital admissions in patients with dilated cardiomyopathy undergoing dialysis [44]; and spironolactone to suppress the production of aldosterone, which stimulates ventricular hypertrophy [45]. The use of statins in the UC population has shown mixed results, appearing to vary depending on the specific patient group under study.

Regardless of pharmacotherapy, the eventual treatment for UC in most cases is renal replacement therapy, which can be accomplished by hemodialysis, peritoneal dialysis, or renal transplantation.

Hemodialysis has been shown in many studies to potentiate reverse cardiac remodeling and to reverse some of the clinical sequelae of UC [46]. This is perhaps the most important treatment for UC in our current armamentarium.

Peritoneal dialysis is a home therapy that may represent a preferable treatment option for some patients versus hemodialysis, as it does not require patients to travel 3–5 times a week to a dialysis center and may even be carried out while the patient is sleeping. Costs may also be lower than those for dialysis, depending on the treatment setting. However, there may be greater risk of treatment discontinuation with peritoneal dialysis because it is self-administered, and some studies suggest that if not adequately monitored, patients undergoing treatment via this modality appear to have worse CV outcomes, including increased LV mass index, in the long term (beyond 2 years of dialysis treatment) [47].

Finally, renal transplantation confers a significant survival advantage versus other treatment modalities, as well as long-term improvement in LVEF and reduction in LVH [48]. These advantages must be balanced with difficulties in finding donors (and risks posed by potential wait times), the cost of the procedure, and the risk of perioperative adverse events. Palliative/supportive treatment options for UC patients to date do not differ from those for heart failure patients in general; this treatment should be per accepted guidelines unless further evidence emerges that is specific to this patient group [49, 50].

Beyond these existing treatments, new and experimental therapies offer the potential for superior outcomes. Among these is a potential role for salvianolic acid B (Sal B), the active component in *Salvia miltiorrhiza Bunge* (red sage), which is widely used as an herbal medication in traditional Chinese medicine. A recent study using a rat model of UC (which also used speckle-tracking echocardiography) reported efficacy in reducing cardiac hypertrophy, edema, inflammation, and fibrosis [51].

The role of recombinant human erythropoietin (EPO) administration in the treatment of anemia in CKD has been explored with controversial results. Initial studies in anemic patients undergoing dialysis showed improved quality of life and reduced need for transfusions; the use of erythropoiesis-stimulating agents (ESAs) subsequently became widespread. However, some recent studies have indicated an increased risk of stroke without a survival benefit [52]. It appears that while EPO may be necessary for some anemic patients with CKD, clinicians should be aware of the potential for adverse events.

The role of vitamin D supplementation in this population is also being explored, with some positive findings in terms of LV fiber shortening [53]. Intravenous iron supplementation has also shown utility in patients with CKD undergoing dialysis who have low ferritin concentrations, resulting in fewer adverse CV events and lower risk of death [54].

## Future directions

Further research is clearly needed to address the gaps in the literature regarding the etiology, diagnosis, and management of UC. This section describes the shortcomings in the current understanding of UC and offers suggestions for the types of prospective or retrospective research that could be carried out to address these gaps. There is an overall lack of research in this population in general; for example, many trials of therapies aimed at reducing CV risk have systematically excluded patients with CKD. As a result, there is a relative paucity of specific data, with much research focusing either on all patients with CKD or all those with heart failure.

A promising area for research is that related to the cardiac effects resulting from the interplay of FGF23 and Klotho [55]. If this relationship can be further illuminated, the balance of these factors presents an appealing therapeutic target.

The contributions of inflammation and systemic oxidant stress should be further explored, especially as oxidative stress appears to play some role in the molecular pathways postulated to be associated with the development of UC via myocyte hypertrophy and DIF causing expansion of interstitial spaces [12].

Besides these specific factors, research should be aimed at improving detection and treatment and at addressing the limitations of current methods, as noted above. There is a gap between the understanding of the underlying pathomechanisms and the development of actual treatments (including pharmacotherapy). In other words, we can diagnose the disease, but there are virtually no specific treatment options for UC. The potential and emerging modalities identified in Table 2 present opportunities that, with further research, could bring effective treatments to fruition. There also exists a gap when it comes to diagnosis: Early diagnosis is difficult because UC frequently overlaps with ischemic cardiomyopathy, hypertensive cardiomyopathy, and diabetic cardiomyopathy as a result of their common risk factors. Given the burden of this disease and its poor outcomes, future research should focus on differentiating UC from other types of cardiomyopathies in terms of identifying specific risk factors, quantifying risk, and optimizing treatment. Researchers are urged to publish their findings regardless of the success of diagnostic and treatment approaches.

## Conclusions

The research to date has been essential in forming the modern understanding of the etiology, pathogenesis, diagnosis, and treatment of UC. However, this improved understanding has not yet led to applicable modifications to practice for the treating clinician, apart from substantial improvements in imaging technology. Innovative research must take place to fill these gaps in current research. Differential diagnosis, risk quantification and stratification, and specific treatment options for UC are important areas where research must transform our current understanding into applicable tools for the clinician. Clinicians, institutions, and organizations must work toward furthering the science and acting on it to improve patient outcomes in this disease that has such a significant global burden.

## Data Availability

Not applicable.
